# A fossil biting midge (Diptera: Ceratopogonidae) from early Eocene Indian amber with a complex pheromone evaporator

**DOI:** 10.1038/srep34352

**Published:** 2016-10-04

**Authors:** Frauke Stebner, Ryszard Szadziewski, Peter T. Rühr, Hukam Singh, Jörg U. Hammel, Gunnar Mikalsen Kvifte, Jes Rust

**Affiliations:** 1 Steinmann-Institut, Abteilung Paläontologie, Nussallee 8, 53115 Bonn, Germany; 2 University of Gdańsk, Department of Invertebrate Zoology and Parasitology, Wita Stwosza 59, 80- 308 Gdańsk, Poland; 3 Zoologisches Forschungsmuseum Alexander Koenig, Zentrum für Molekulare Biodiversitätsforschung, Adenauerallee 160, 53113 Bonn, Germany; 4 Birbal Sahni Institute of Palaeosciences, 53 University Road, Lucknow, India; 5 Helmholtz-Zentrum Geesthacht, Institut für Werkstoffforschung, Max-Planck-Str. 1, 21502 Geesthacht, Germany; 6 University Museum of Bergen, Department of Natural History, P.O. Box 7800, University of Bergen, 5040 Bergen, Norway; 7 Universität Kassel, Institut für Biologie, Fachgebiet Limnologie, Heinrich-Plett-Straße 40, 34132 Kassel-Oberzwehren, Germany

## Abstract

The life-like fidelity of organisms captured in amber is unique among all kinds of fossilization and represents an invaluable source for different fields of palaeontological and biological research. One of the most challenging aspects in amber research is the study of traits related to behaviour. Here, indirect evidence for pheromone-mediated mating behaviour is recorded from a biting midge (Ceratopogonidae) in 54 million-year-old Indian amber. *Camptopterohelea odora* n. sp. exhibits a complex, pocket shaped structure on the wings, which resembles the wing folds of certain moth flies (Diptera: Psychodidae) and scent organs that are only known from butterflies and moths (Lepidoptera) so far. Our studies suggests that pheromone releasing structures on the wings have evolved independently in biting midges and might be much more widespread in fossil as well as modern insects than known so far.

Mating in insects is a complex behavioural trait involving varying combinations of visual, tactile, acoustic, and olfactory signals that even in modern insects are only poorly understood. Unsurprisingly, direct evidence of behavioural traits in the fossil record is fragmentary, difficult to detect and requires exceptional fossilisation conditions. In amber, mating behaviour has principally been observed by direct evidence from specimens preserved in copula, or by numerous conspecific syninclusions indicating swarm behaviour^1^. However, evidence of highly specialized courtship communication behaviour such as the use of pheromones is extremely rare in the fossil record and only reported from single case studies^2^.

Sex attractant pheromone production in modern Ceratopogonidae has been studied primarily in taxa of importance for human health or domestic animals. In blood feeding species of *Culicoides* volatile as well as aphrodisiac contact pheromones have been recorded^3^,^4^,^5^. In *Culicoides nubeculosus* and *C*. *melleus* contact pheromones are involved in mating stimulation and precopulatory orientation^4^,^6^. In both species the female abdomen is thought to be the site of pheromone emission^7^.

Here a striking pocket-like structure is described on the wings of a female of a new species of the ceratopogonid genus *Camptopterohelea* Wirth and Hubert, 1960 reported from early Eocene Indian amber. The highly specialised structure is unknown among all fossil and extant Ceratopogonidae and shows evidence for the emission of sex attractant pheromone.

**Systematic palaeontology**

Family Ceratopogonidae Newman, 1834

Subfamily Ceratopogoninae Newman, 1834

Genus *Camptopterohelea* Wirth & Hubert, 1960

**Type species:** *Camptopterohelea hoogstraali* Wirth & Hubert, 1960

**Generic diagnosis:** Features for females based on Wirth & Hubert (1960) and Wirth & Wada (1979): Eyes broadly separated; palpus 3-segmented; single and moderately long claws on all legs; wing greatly modified, costa short, second radial cell small, media and r-m crossvein absent, anterior portion of wing with transverse area with dense, coarse microtrichia, macrotrichia absent. **Distribution** Five extant species in the Oriental Region: India, Indonesia, Philippines, Malaysia.

***Camptopterohelea odora*** **n. sp.**

(Figure 1a–d, Supplementary Figs 1a–d and 2, Supplementary video).

**Holotype female:** Tad-859a, in amber from Gujarat State, western India, Tadkeshwar lignite mine, Cambay Formation (lowermost Eocene, Ypresian^8^,^9^,^10^); deposited in the collection of the American Museum of Natural History, New York.

**Diagnosis:** Wing with spherical pocket-shaped structure at proximal third of costa; medial veins and CuA_1_ not apparent.

**Description:** Female Body length: 0.9 mm.

Head: Eyes separated. Flagellum (Supplementary Fig. 1a) with 13 flagellomeres, length: 0.375 mm, antennal ratio (AR) 0.85; flagellomeres 2–10 of similar length, flagellomeres 11 and 12 each about 1.5x as long as flagellomere 10, terminal flagellomere longest, twice as long as flagellomere 10; sensilla coeloconica not visible, some proximal and distal flagellomeres with stout, peg like sensilla trichodea. Proboscis short. Palpus (Fig. 1b, Supplementary video) very short, with 4 clearly visible palpal segments. Whether number of palpal segments is reduced from the “primitive” condition of 5 segments to 4 segments (i.e., whether “primitive” first or second palpal segment is reduced/absent) or whether the specimen has 5 palpal segments but the basal one is simply not visible remains unclear. Second visible segment (the antepenultimate and therefore “primitive” third segment) swollen, with roundish structure that might be a sensory pit or an artefact.

Wings (Fig. 1a–d): Right wing basally twisted 180° so ventral surface is directed dorsally, distal half of wing folded backwards on itself; left wing broad, length: 0.62 mm, greatest width: 0.35 mm; radial and median veins reduced, CuA_1_ reduced, CuA_2_ well developed; wing membrane covered with distinct microtrichia. Proximal third of costa at upper wing surface with strongly sclerotized dark brown spherical pocket that opens on lower wing surface (Fig. 1a–d, Supplementary Fig. 2, Supplementary video); more than half of pocket’s opening covered by a wing membrane fold from the upper surface, apical part of fold covered by fringe of dense setae or spicules. Interior of pocket with globular structure. Small nodule-like structure basal to pocket, connected to pocket by vein-channel (Fig. 1c,d, Supplementary video).

Thorax: Length: 0.37 mm, greatest width: 0. 23 mm. Legs (Supplementary Fig. 1b–d) slender; each armed with single long claw; fourth tarsomeres slightly cordiform; tarsal ratio of fore leg TR(I) 2.5, mid leg TR(II) 2.8, hind leg TR(III) 3.7.

Abdomen: Distorted, dorso-ventrally shrunken, with short cercus.

Remarks: The fossil species is known only from a single female. Due to the position of the specimen in the stone and the quality of the amber matrix light optical investigation of *Camptopterohelea odora* n. sp. was limited and descriptions of some characters are based on 3D reconstruction from synchrotron micro-computed tomography scans (SRμCT) only. Single ommatidia are not clearly visible in the reconstruction so that the distance between the eyes in *C*. *odora* n. sp. cannot be measured exactly. The number of palpal segments is given with “at least four” since the presence of a further (basal) segment cannot be stated without doubt. Although position and shape of the roundish structures on the swollen palpal segment are in accordance with sensory pits of modern species of *Camptopterohelea* it cannot be ruled out that they are artefacts.

Discussion: The specimen belongs to the subfamily Ceratopogoninae Newman, 1834 based on the following combination of characters: apical flagellomere with blunt tip, flagellomeres not sculptured; claws gently curved, empodium vestigial; female cercus short.

The fossil is assigned to the genus *Camptopterohelea* Wirth & Hubert, 1960 based on the combination of the following female characters: claws long and single; wing membrane without macrotrichia, costa short, media and r-m crossvein absent. *Camptopterohelea* belongs to a group of closely related genera (further comprising *Cacaohelea* Wirth & Grogan, 1988; *Eohelea* Petrunkevich, 1957; *Parastilobezzia* Wirth & Blanton, 1970)^11^. Further diagnostic female characters that assign *C*. *odora* to *Camptopterohelea* and distinguish it from *Cacaohelea*, *Eohelea* and *Parastilobezzia* are listed in Supplementary Table 1.

*Camptopterohelea odora* n. sp. differs from all remaining species of this genus in the higher number of palpal segments (at least four in *C*. *odora* n. sp. compared to three). This is regarded a plesiomorphic condition relative to extant species, which have the primitive first two palpal segments reduced. Furthermore, *C*. *odora* shows the most reduced wing venation with the radial cells completely absent. Extant representatives of *Camptopterohelea* either have two radial cells with the second being very small (*C*. *hoogstraali* Wirth & Hubert, 1960; *C*. *javanensis* Wirth & Wada, 1979; *C*. *tokunagai* Wirth & Wada, 1979), or only one small radial cell (*C*. *admirabilis* Das Gupta & Sarkar, 1982; *C*. *distincta* Das Gupta & Sarkar, 1982). Due to the position of the pocket on the wing, which roughly corresponds to the position of the radial cells in extant species, and the fact that the radial veins in extant species of *Camtopterohelea* are swollen, it is likely –though not definitely provable- that the pocket is formed by the radial veins. It is believed that this is an apomorphic condition, which has not been developed in all other known species. However, it cannot be ruled out that this character has been reduced in extant species.

In accordance with the systematic description and discussion above the generic diagnosis for *Camptopterohelea* should be changed in part as follows: antepenultimate palpal segment broadly swollen; wing with short costa (CR 0.56 or less), without media and r-m crossvein, radial cells showing various stages of reduction: both radial cells present but second very short, only one radial cell present, both radial cells absent (*C*. *odora* n. sp.).

## Discussion

Within the Ceratopogoninae, a sister group relationship has been proposed for the genera *Camptopterohelea*+*Eohelea* and *Cacaohelea*+*Parastilobezzia* based on the presence of a distinctive patch of elongate microtrichia on the female’s wing^11^. This character is present as a small patch of somewhat thicker microtrichia at the wing apex in *Parastilobezzia* Wirth & Blanton, 1970, as a single darkened, nearly circular patch of elongate microtrichia in *Cacaohelea* Wirth & Grogan, 1988, and a variably structured oval patch below the apex of the second radial cell in the fossil genus *Eohelea* Petrunkevitch, 1957.

Five Recent species of the genus *Camptopterohelea* have been recorded in the Oriental Region^12^,^13^,^14^ in all of which females have a reduced wing venation with thickened radial veins R_2+3_ (Supplementary Fig. 3a,b). These veins form a distinct and variously developed second radial cell. Beyond the apex of the radial cells there is a patch of distinctive microtrichia, which is associated with the vein M at least in *C*. *javanensis* and *C*. *admirabilis* and situated on a dorsally raised area in *C*. *admirabilis* and in a depression dorsally in *C*. *javanensis*. Further to this, SEM imaging of the wing of an undescribed *Camptopterohelea* female from Malaysia reveals a patch of stubby, broken microsetae inside the radial cell (Supplementary Fig. 4a–c).

*C*. *odora* n. sp. lacks a distinctive patch of microtrichia but exhibits the most reduced wing venation among all species with the radial veins modified into a wing pocket. Based on our knowledge of the Recent species *C*. *distincta* the wing pocket of *C. odora* is probably sexually dimorphic and may be a more developed modification of the reduced and swollen radial cell on the wings of the two Recent Indian species^14^. It shows morphological similarities with the scent organs on wings of certain taxa of Lepidoptera and is believed to have a similar function: the storage and dissemination (an evaporator) of sex attractant pheromones.

Sex pheromones play an important role in insect courtship. Generally there are two types of pheromones: volatile ones that act at a distance and non-volatile ones that act on contact. In many insects pheromones are synthesized in specialized epidermal gland cells^15^. In some cases however, like in tiger moths, pheromones are produced in oenocytes and transported to the releasing organ by the haemolymph^16^. The mechanisms for dissemination of pheromones are minimally studied but in some cases release seems to take place by diffusion through pores^4^,^15^. Dispersal of volatile pheromones from the surface of the cuticle has been proposed to be facilitated by surrounding microtrichia^17^. Wing vibration, which often accompanies courtship behaviour, seems to act as a fan for distributing the pheromones, not only in taxa with pheromone releasing structures positioned on the wing. In the biting midge *Culicoides nubeculosus* Meigen, 1830, for example air flow and wing flutter are required for the dispersion of a pheromone released by the abdomen^18^.

In Diptera the most similar structures to the wing pocket of *C*. *odora* n. sp. can be found in moth flies (Diptera: Psychodidae). Males of the psychodid *Ulomyia fuliginosa* Meigen, 1804 have a pocket-like pouch on each wing, which opens on the lower surface (Supplementary Fig. 5a,b). Although pheromone production in males by eversible mesothoracic scent organs has been proven in *Ulomyia*^19^ a pheromone related function of the pouch has not been taken into consideration. In the fossil psychodid genus *Succinarisemus* Wagner, 2001 males have an enlarged, probably eversible area at the costal region of the wing, which is thought to be involved in courtship behaviour^20^,^21^ (Supplementary Fig. 6a,b), and very similar to the scent organs on the wings of some Lepidoptera. In the lepidopteran *Hydrillodes* Guenée, 1854 males have a large costal fold on the upper side of each forewing (Supplementary Fig. 7b). In closed position these scent organs can be recognized as a semicircle flap forming a pocket (Supplementary Fig. 7a). Location of the scent-disseminating organs in pockets or cuticular folds is presumably important for retaining the scent from evaporation^22^. In some Lepidoptera release of scents by the wings takes place only after breakage of certain scent scales (androconia), which have predetermined braking points^22^.

Whether the wing patches of broken microsetae inside the radial cell of the Recent *Camptopterohela* female are involved in pheromone release remains open. If so, however it seems likely that the wing pocket of *C*. *odora* n. sp., which is most probably formed by the radial veins, represents a much more derived modification of this scent releasing structure. Based on morphological investigation we propose the following scenario for pheromone production and dissemination in the fossil *C*. *odora* n. sp.: the wing pocket serves as a reservoir that stores volatile pheromones and prevents the pheromones from unregulated and accidental evaporation. Small droplets of liquid pheromone are absorbed by the setae covering the opening of the pocket and released for mate attraction by wing fanning or during flight. Whether pheromones are produced by scent glands inside the pocket or transported into the pocket from the small nodule in front of the pocket, or from other regions of the thorax through wing veins, remains uncertain. The connection of the smaller nodule with the wing pocket by a channel makes the nodule as a place of synthesis plausible. A similar pheromone mechanism has been described for *Eldana saccharina* (Lepidoptera), where inside the costal vein a complex glandular structure is situated and from which the secretory products flow in ducts into the lumen of a canal on the lower side of the wing^23^. The proposed scenario implies that in *C*. *odora* n. sp. long distance volatile pheromones are used, which would be related to mating behaviour. In many Ceratopogonidae the basic mating system is a swarm in which males recognize females by their wing beat frequency. Interestingly, *Camptopterohelea* males have antennae without the plume of long setae, and a reduced number of flagellomeres, which suggests a loss of sound perception. This situation is often accompanied by a substrate-based mating system^24^ where recognition of conspecific females has to take place by olfactory, contact and/or visual stimuli.

Visual recognition of females has been suggested by^25^ for species of the fossil genus *Eohelea* from Eocene Baltic and Sakhalin amber. Here, males also have antennae without a plume and females have a variably structured oval patch on the wing. The patch is either composed of ridges with perpendicular micro-ridges or of a honey-comb structure similar to a dipteran’s compound eye, and has been interpreted to be light reflecting. A possible pheromone disseminating function of the wing patches has not been taken into consideration for *Eohelea* so far. Since mating and courtship behaviour is a complex trait with several stimuli working in concert visual mate recognition might not be ruled out for *C*. *odora* n. sp. However, eyes in males of *Camptopterohelea*, as in *Eohelea*, are small and unmodified suggesting that they are not adapted to play an important role in mating.

Our findings show that pheromone dispersion from the wings of *C*. *odora* is strongly indicated from a morphological and behavioural point of view and is more widespread in Diptera than so far assumed.

## Material and Methods

### Morphological terms

The special morphological terms and abbreviations used in the paper follow those explained by refs 26 and 27. The antennal ratio (AR) is obtained by dividing the combined length of the distal five flagellomeres by the combined length of the proximal eight flagellomeres; the tarsal ratio (TR) is obtained by dividing the length of the basitarsus by the length of the second tarsomere; the costal ratio (CR) is calculated by dividing the length of the costa by wing length.

### Amber material

**Figure 1:**
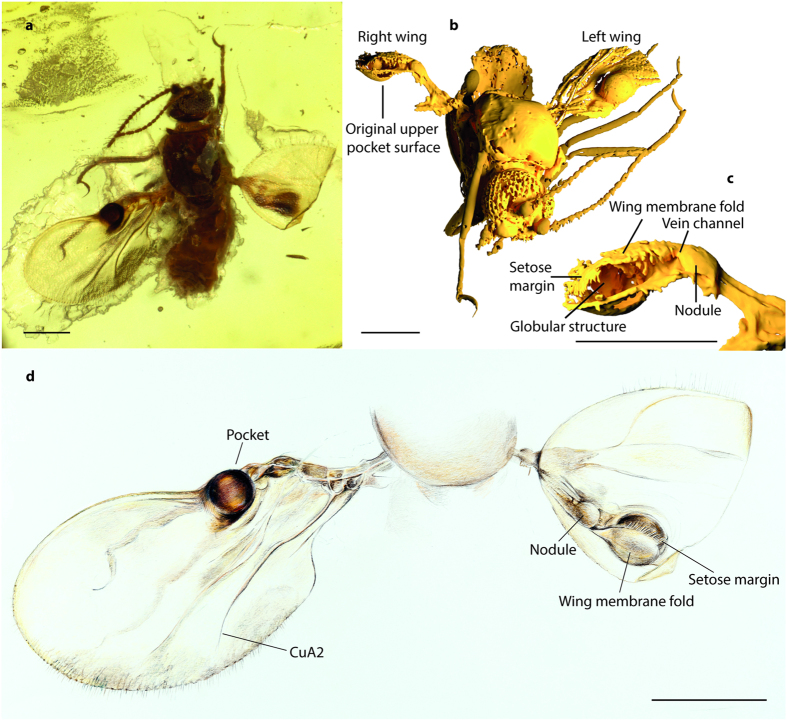
*Camptopterohelea odora* n. sp. (**a**) Photograph of holotype Tad-859a in dorsal view after the first embedding process. Scale bar: 0.2 mm. (**b**) 3D reconstruction from SRμCT scans. Membranous parts only partly reconstructed for left wing and not reconstructed for right wing. The right wing is basally twisted 180° so the ventral surface is directed dorsally. Scale bar: 0.2 mm. (**c**) 3D reconstruction of right wing pocket from SRμCT scans. Scale bar: 0.2 mm. (**d**) Drawing of wings and posterior portion of the thorax of *Camptopterohelea odora* n. sp. in dorsal view. Left wing with pocket visible from the upper side, right wing twisted 180° at base with ventral surface of pocket visible from the lower side (apical half of wing folded back on itself). (Figure 1d by Dorothea Kranz, Steinmann Institut, Bonn). Scale bar: 0.2 mm.

The holotype of *Camptopterohelea odora* n. sp. is deposited in the collection of the American Museum of Natural History (AMNH), New York (coll. no. Tad-859a). Due to brittleness the piece of amber has been embedded in artificial resin (Araldite 2020 A/B, Huntsman) prior to preparation. Afterwards the sample has been ground using a Buehler “Phoenix Beta” grinding machine with Sic Grinding paper of grades 120, 300, 1000, 2500 and 4000 (Fig. 1a displays specimen at this point of preparation), followed by re-embedding in artificial resin (Araldite 2020 A/B, Huntsman). During the last step stress cracks in the amber matrix appeared, which result in a slightly limited view of the specimen (see Supplementary Fig. 8).

For the taxonomic identification and investigation a “Zeiss Discovery V8 Stereomicroscope” was used. Drawings were rendered with the aid of a drawing tube and performed with “Adobe Illustrator CS2”. Measurements are given in millimetres. Photographs of the amber inclusion were made with an “AXIO Zoom.V16 Stereomicroscope” (Carl Zeiss, Jena) equipped with an “AXIOCam HRc Digital Camera” (Zeiss), using the “extended depth of focus” function.

*Succinarisemus totolapensis* Coty *et al*., 2013 in amber from Totolapa, Chiapas, Mexico, Holotype No. 104 (D. Coty collection), was borrowed from the Senckenberg Forschungsinstitut und Naturmuseum, Frankfurt am Main, Germany. Photographs were made with an “AXIO Zoom.V16 Stereomicroscope” (Carl Zeiss, Jena) equipped with an “AXIOCam HRc Digital Camera” (Zeiss), using the “extended depth of focus” function.

### Recent material

The *Camptopterohelea* specimen (Supplementary Fig. 4a–c), collected in the Kipandi Butterfly Park, Sabah, Malaysia, on loan to Art Borkent (Royal British Columbia Museum, Columbia, Canada), belongs to the collection of the California State Collection of Arthropods, California Department of Food and Agriculture, Sacramento, USA. The wing was prepared for scanning electron imaging (XL30S, FEG, Phillips, Eindhoven) and scanned with an acceleration voltage of 3 kV.

*Ulomyia fuliginosa* specimens were sampled in Alt-Lübars, Berlin, Germany. The wing was prepared for scanning electron imaging (CamScan MV 2300) and scanned with an acceleration voltage of 20 kV.

*Hydrillodes* specimens, catalogue no. 88/210, collected in the Philippines, were borrowed from the collection of the Zoologisches Forschungsmuseum Alexander Koenig, Bonn, Germany. Photographs were made with a “Nikon D3x” Camera with an “AF-S Micro-NIKKOR 60 mm 1:2,8 G ED” lens.

### Synchrotron radiation based X-ray micro-computed tomography

For synchrotron micro-computed tomography (SR-μCT) *C. odora* was mounted on a specimen holder and scanned at the Deutsches Elektronen Synchrotron (DESY; beamline IBL P05 at PETRA III, operated by the Helmholtz-Zentrum Geesthacht, Hamburg, Germany) with a stable beam energy of 10 keV and attenuation contrast mode^28^,^29^,^30^. The 5x lens provided a field of view of 7.4 × 7.4 mm, resulting in an effective pixel size of 2.4 × 2.4 μm. SR-μCT of the specimen resulted in a distinct darkening of the amber matrix in the scanned area (for comparison see Supplementary Fig. 1a and Supplementary Fig. 8). Subsequent manual segmentation and semi-automatic segmentation with the ‘region competition’ algorithm was done in ITK-SNAP^31^. Vertex reduction of the resulting surface meshes was accomplished by using the ‘Quadric Edge Collapse Decimation’ function of MeshLab (http://meshlab.sourceforge.net). In Blender (http://blender.org) the objects where slightly smoothed using the ‘Smooth’ modifier, preserving as much detail as possible. Final rendering of the figures and the Supplementary video was also done in Blender. All software packages are freely distributed under the General Public License (GPL).

## Additional Information

**How to cite this article**: Stebner, F. *et al*. A fossil biting midge (Diptera: Ceratopoginidae) from early Eocene Indian amber with a complex pheromone evaporator. *Sci. Rep.* **6**, 34352; doi: 10.1038/srep34352 (2016).

## Supplementary Material

Supplementary Information

Supplementary Video
