# Corrigendum: A fossil biting midge (Diptera: Ceratopogonidae) from early Eocene Indian amber with a complex pheromone evaporator

**DOI:** 10.1038/srep41899

**Published:** 2017-03-08

**Authors:** Frauke Stebner, Ryszard Szadziewski, Peter T. Rühr, Hukam Singh, Jörg U. Hammel, Gunnar Mikalsen Kvifte, Jes Rust

Scientific Reports 6: Article number: 34352; 10.1038/srep34352 published online: 10 04 2016; updated: 03 08 2017.

This Article was not registered at Zoobank prior to publication. The following ‘Systematic palaeontology’ and ‘Nomenclatural Acts’ subsections have been added to the Material and Methods section.

**Systematic palaeontology**

Family Ceratopogonidae Newman, 1834

Subfamily Ceratopogoninae Newman, 1834

Genus Camptopterohelea Wirth & Hubert, 1960

**Type species**: Camptopterohelea hoogstraali Wirth & Hubert, 1960

**Generic diagnosis**: Features for females based on Wirth & Hubert (1960) and Wirth & Wada (1979): Eyes broadly separated; palpus 3-segmented; single and moderately long claws on all legs; wing greatly modified, costa short, second radial cell small, media and r-m crossvein absent, anterior portion of wing with transverse area with dense, coarse microtrichia, macrotrichia absent.

**Distribution**: Five extant species in the Oriental Region: India, Indonesia, Philippines, Malaysia.

**Camptopterohelea odora n. sp.**

urn:lsid:zoobank.org:act:711D1AA0-326F-4326-8EB3-E96D555C3CC6

**Nomenclatural Acts**

The electronic edition of this article conforms to the requirements of the amended International Code of Zoological Nomenclature, and hence the new names contained herein are available under that Code from the electronic edition of this article. This published work and the nomenclatural acts it contains have been registered in ZooBank, the online registration system for the ICZN. The ZooBank LSIDs (Life Science Identifiers) can be resolved and the associated information viewed through any standard web browser by appending the LSID to the prefix “http://zoobank.org/”. The LSID for this publication is: urn:lsid:zoobank.org:pub:BF9E1432-6279-49B6-8334-DD9BBF052B48. The electronic edition of this work was published in a journal with an ISSN, and has been archived and is available from the following digital repositories: PubMed Central, LOCKSS.

An earlier edition of this work published electronically on 4 October 2016 did not fulfill the requirements for electronic publication under the amended ICZN Code.

