# Supplementary material for: A fossil biting midge (Diptera: Ceratopogonidae) from early Eocene Indian amber with a complex pheromone evaporator
Source: Sci Rep. 2016 Oct 4;6:34352. doi: 10.1038/srep34352 (PMC5048152; doi:10.1038/srep34352)
Supplement: Supplementary Information [file srep34352-s1.pdf]

A fossil biting midge (Diptera: Ceratopogonidae) from early Eocene Indian amber with a complex pheromone evaporator

Frauke Stebner, Ryszard Szadziewski, Peter T. Rühr, Hukam Singh, Jörg U. Hammel, Gunnar Mikalsen Kvifte, Jes Rust

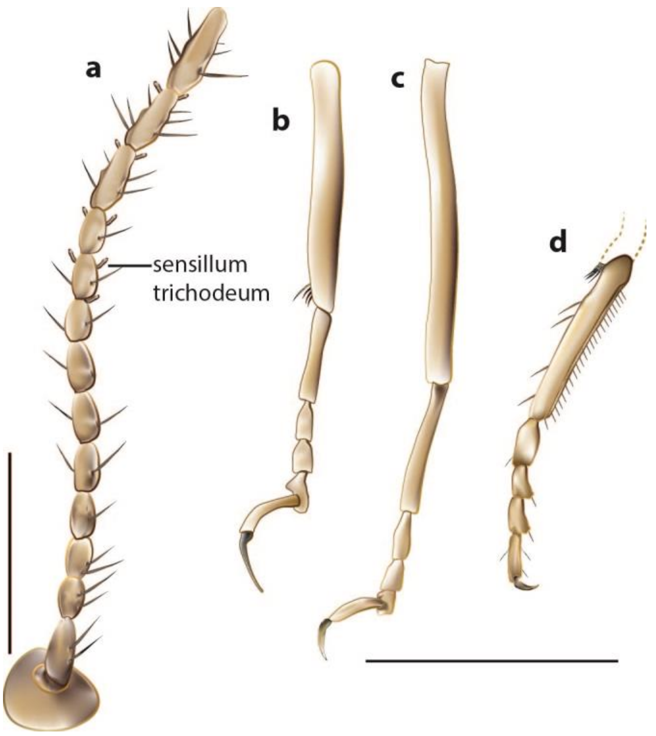

Supplementary Figure 1. Drawings of antenna and legs of *Camptopterohelea odora* n. sp. a) Pedicel and flagellomeres 1-13. b) Tibia and tarsomeres 1-5 of fore leg. c) Tibia and tarsomeres 1-5 mid leg. d) Distal part of tibia and tarsomeres 1-5 of hind leg. Scale bar antenna: 0.1 mm, scale bar legs: 0.2 mm.

(Supplementary Figures 1a-d by Dorothea Kranz, Steinmann Institut, Bonn).

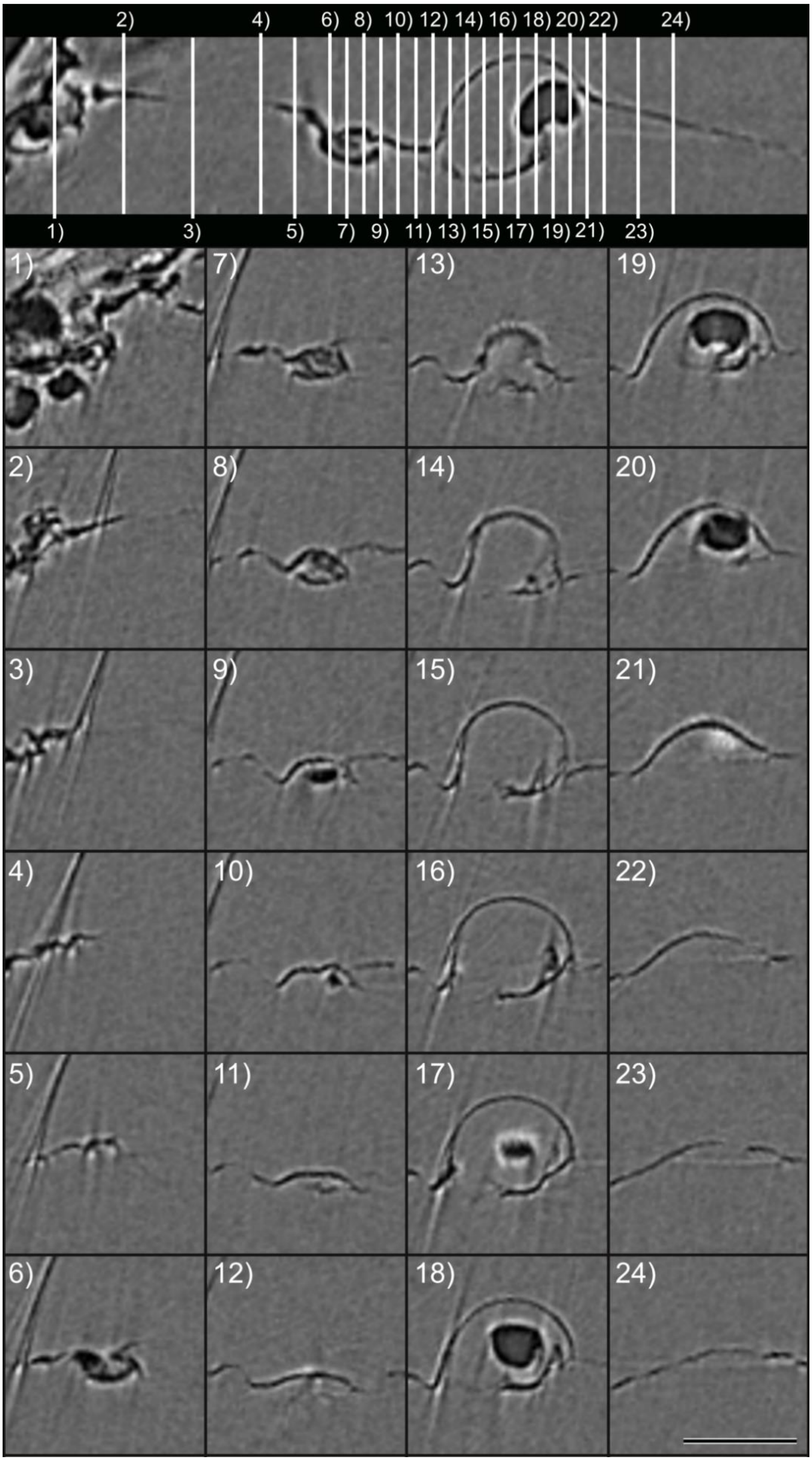

Supplementary Figure 2. Successive transversal SR-µCT sections through the left wing pocket of *Camptopterohelea odora* n.sp. Scale bar: 0.1 mm.

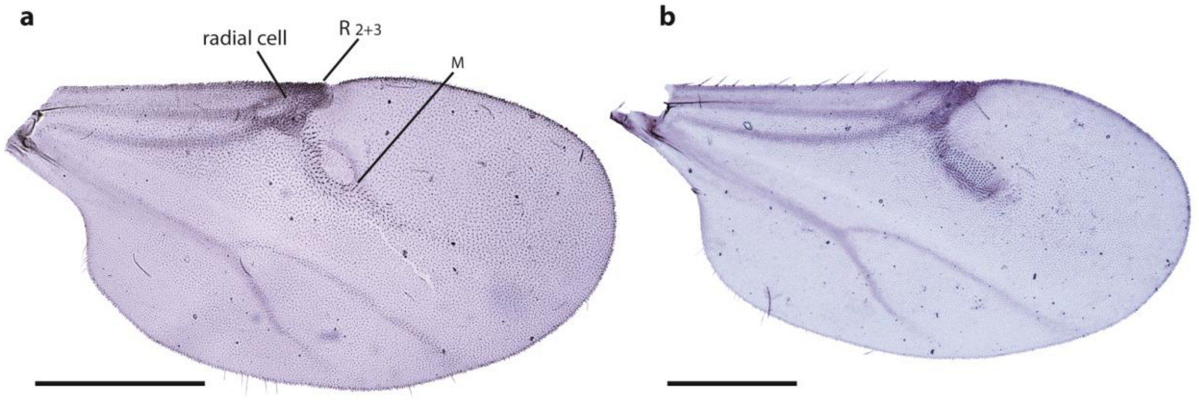

Supplementary Figure 3. Photograph of wings of Recent *Camptopterohelea* females. a) *Camptopterohelea javanensis*. Scale bar: 0.2 mm. b) *Camptopterohelea admirabilis*. Scale bar: 0.2 mm. (Photography by Art Borkent, Canada).

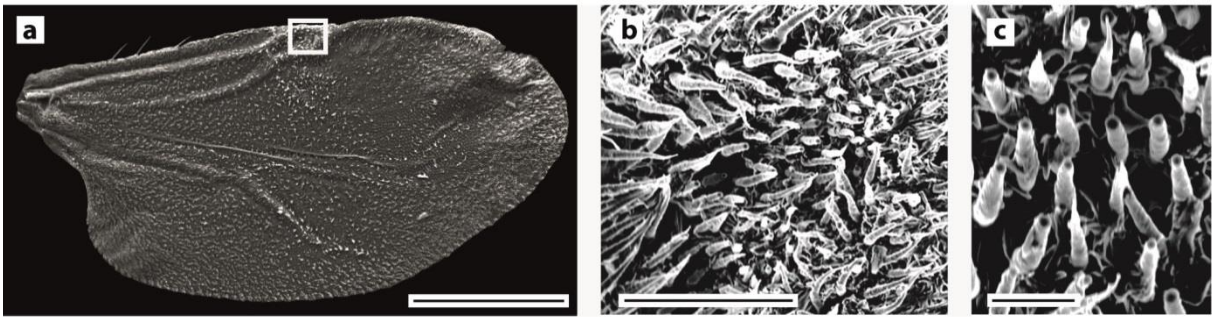

Supplementary Figure 4. SEM image of a wing of a Recent undescribed *Camptopterohelea* female from Malaysia. a) Complete wing with white frame marking the apex of the radial cell and detail image b. Scale bar: 0.2 mm. b) Detail image of patch with stubby, broken microsetae inside the radial cell. Scale bar: 10 µm. c) Detail image of broken microsetae. Scale bar: 2 µm. (Supplementary Figure 4a by Georg Oleschinski, Steinmann Insitut Bonn; Supplementary Figures 4b, c by Gregor Kirfel, Institut für Zellbiologie, Bonn).

Similar structures in other insect taxa

The wing pockets of *Camptopterohelea odora* n. sp. described here are unknown in all other extant and fossil Ceratopogonidae. Morphologically similar structures in dipterous flies are only known in members of the Psychodidae (moth flies) while the most similar structure outside the Diptera is found in the Lepidoptera.

Diptera

Males of the moth fly *Ulomyia fuliginosa* (Diptera: Psychodidae) have two pairs of eversible mesothoracic pheromone releasing scent organs (mesothoracic appendages after [1]) and a pouch on each wing at the basal portion between the radial and medial veins which opens on the lower wing surface (Supplementary Figs 5a, b).

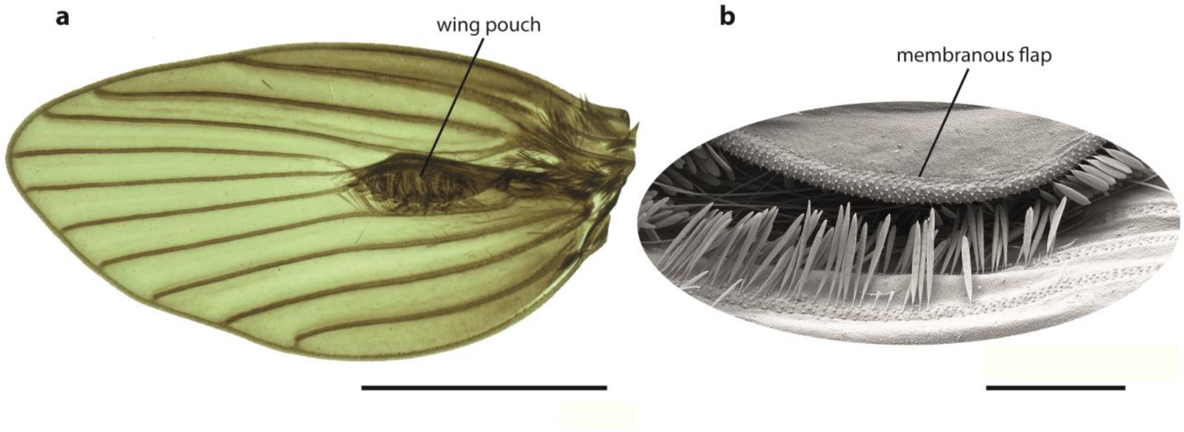

Supplementary Figure 5. Wing of male *Ulomyia fuliginosa*. a) Lower side view showing the pouch. Scale bar: 1 mm. b) SEM detail image of the wing pouch showing the opening of the pouch with the membranous flap that covers the pouch and the wing membrane (bottom), which is involved in the formation of the pouch. Margins of the pouch are covered with scales that stand in sockets over veins. Scale bar: 0.2 mm. (Supplementary Figure 5b by Georg Oleschinski, Steinmann Insitut Bonn).

Close range courtship behaviour of the male includes wing fanning in front of the female, extrusion of the scent organs, and holding one wing over the female's head [2, 1]. The latter phase of the courtship ritual has been interpreted as a tactile stimulus [2] or as a position in which the female's antennae with the olfactory organs come close to the male's mesothoracic scent organs [1]. Nevertheless, the structure of *U. fuliginosa's* wing pouch and the position of the opening above the female's head during courtship suggest that it might be related to pheromone storage and release.

In the fossil genus *Succinarisemus* Wagner, 2001 (Diptera: Psychodidae) males have a broadened and probably eversible area on the upper side of the wing [3, 4], covered by different types of hairs and scales (Supplementary Figs 6a, b). The conspicuous similarity to the wing folds of the lepidopteran *Hydrillodes* might be evidence for a scent disseminating function of this fold.

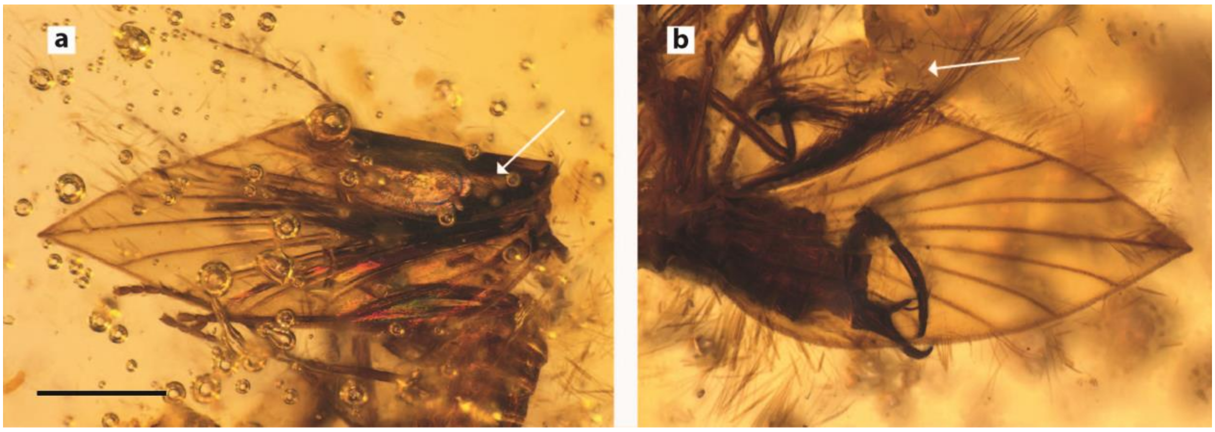

Supplementary Figure 6. Photograph of *Succinarisemus totolapensis* in Mexican amber. a) Broadened area on upper wing margin closed (white arrow). b) Broadened area on upper wing margin open (white arrow). Scale bar: 0.5 mm.

Lepidoptera

In many species of Lepidoptera scent organs are present on different parts of the body, varying from scent scales (androconia) and tuft of hairs to complex, sometimes eversible structures with scent storing and releasing mechanisms [5, 6]. On the wings scent organs can be frequently found in pockets, costal folds or membranous flaps that cover specialized scales and can be everted by wing fanning [6]. In the genus *Hydrillodes* males have a large costal fold on the upper side of the forewing which can be recognized as a semicircle flap in closed position (Supplementary Fig. 7a). Opened out, the scent organ results in a distinct curvature of the wing margin and has different types of hairs and scales inside the fold (Supplementary Fig. 7b).

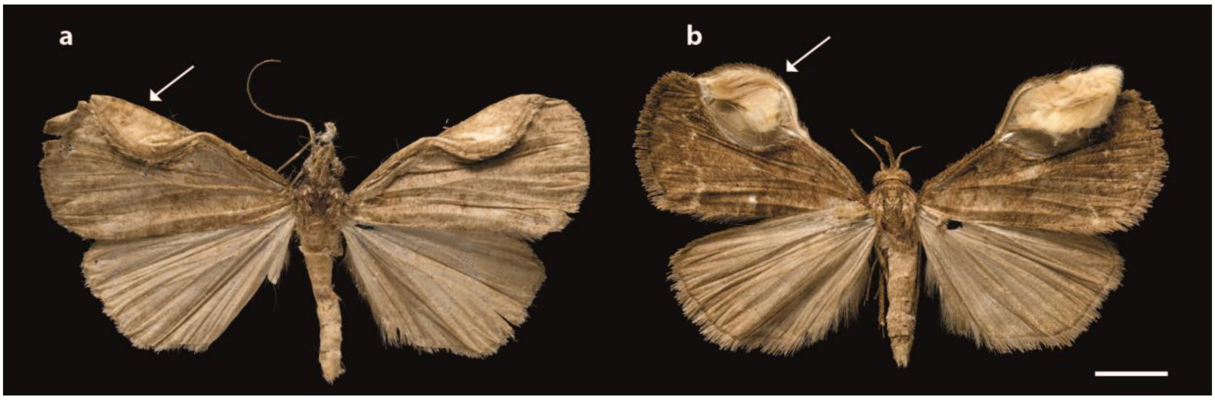

Supplementary Figure 7. Photograph of *Hydrillodes* sp. male. a) Dorsal view with scent organs on upper wing margin closed (white arrow). b) Dorsal view with scent organs on upper wing margin open (white arrow). Scale bar: 4 mm.

(Supplementary Figures 7a, b by Georg Oleschinski, Steinmann Insitut Bonn).

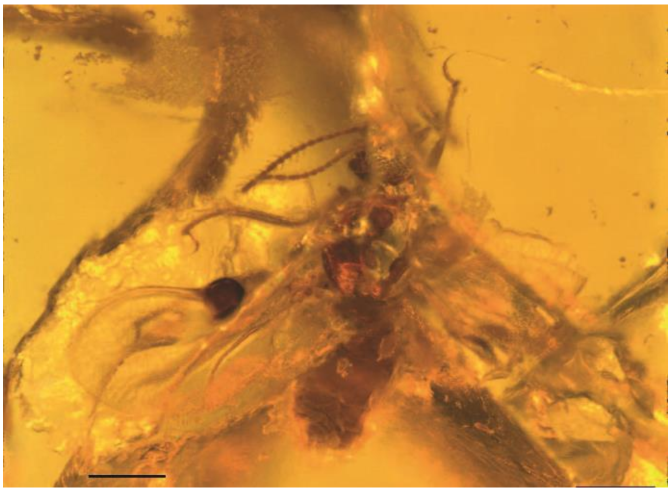

Supplementary Figure 8. Holotype of *Camptopterohelea odora* n. sp. Photograph of Tad-859a in dorsal view after second embedding process and SRµCT with darkening of the amber matrix and stress cracks visible. Scale bar: 0.2 mm.

Supplementary Table 1. Female characters of selected Ceratopogonidae in comparison to set of characters seen in *Camptopterohelea odora* n. sp. Congruent characters of *C. odora* n. sp and extant species of *Camptopterohelea* marked in red.

taxon / character state	*C. odora* n. sp.	*Camptoptero-helea*	*Eohelea*	*Cacaohelea*	*Parastilo-bezzia*
palpus 5 segmented	?		x	x	
palpus 3 segmented		x			x
reduction of palpal segments	if reduced, then 1st or 2nd	1st and 2nd			2nd and 4th
claws long	x	x			x
claws short			x	x	
2 radial cells		x	x	x	x
reduction of radial cells	x (no radial cells present)	x (1 radial cell present)			
costal ratio CR	low (0.34 or less)	low (ca 0.56)	0.9-1	0.7-0.8	ca 1
media present			x	x	x
media absent	x	x			
patch of microtrichia on wing present		x	x	x	reduced
patch of microtrichia on wing absent	x (no patch visible)		x		
r-m present			x	x	x
r-m absent	x	x			
true 3rd palpal segment swollen	x	x			x

Supplementary Video. 3D reconstruction video of *Camptopterohelea odora* n. sp. from SRμCT scans.

References

1. Elger, M. The ultrastructure of special epidermal organs in Psychodidae (Diptera). *Acta Zool.* **62,** 1-16 (1981).

2. Feuerborn, H. J. Der sexuelle Reizapparat (Schmuck-, Duft- und Berührungsorgane) der Psychodien nach biologischen und physiologischen Gesichtspunkten untersucht. *Arch. Naturgesch.* A **88,** 1-137 (1922).

3. Wagner, R. A remarkable new species and genus of moth-flies (Diptera, Psychodidae, Psychodinae) from Dominican amber. *Stud. Dipterol.* **8,** 423-426 (2001).

4. Coty, D., Salamé, Y., Nel, A. & Azar, D. in *Insect Evolution in an Amberiferous and Stone Alphabet* (eds Azar, D. et al.) 13-25 (Brill, Leiden, Boston, 2013).

5. Lödl, M. Details of the "posterior abdominal brush" and other scent organs of quadrifine noctuids with special reference to Hypeninae and Herminiinae (Lepidoptera: Noctuidae). *Quadrifina* **3,** 279-294 (2000).

6. Birch, M. C., Poppy, G. M. & Baker, T. C. Scents and eversible scent structures of male moths. *Annu. Rev. Entomol.* **35,** 25-58 (1990).
